# An enhanced fresh cadaveric model for reconstructive microsurgery training

**DOI:** 10.1007/s00238-018-1414-3

**Published:** 2018-04-25

**Authors:** Tarak Agrebi Moumni Chouari, Karen Lindsay, Ellen Bradshaw, Simon Parson, Lucy Watson, Jamil Ahmed, Alain Curnier

**Affiliations:** 1 Aberdeen University Anatomy Department, The Suttie Centre for Teaching and Learning in Healthcare, Aberdeen, Scotland UK; 2 Plastics and Reconstructive Surgery Department, Aberdeen Royal Infirmary, Aberdeen, Scotland UK; 3 Bristol University Centre for Applied Anatomy, School of Veterinary Science, Bristol, England UK

**Keywords:** Cadaveric training, Surgical training, Hand surgery, Microsurgery, Gelatine injection

## Abstract

**Background:**

Performing microsurgery requires a breadth and depth of experience that has arguably been reduced as result of diminishing operating exposure. Fresh frozen cadavers provide similar tissue handling to real-time operating; however, the bloodless condition restricts the realism of the simulation. We describe a model to enhance flap surgery simulation, in conjunction with qualitative assessment.

**Methods:**

The fresh frozen cadaveric limbs used in this study were acquired by the University. A perfused fresh cadaveric model was created using a gelatin and dye mixture in a specific injection protocol in order to increase the visibility and realism of perforating vessels, as well as major vessels. A questionnaire was distributed amongst 50 trainees in order to assess benefit of the model. Specifically, confidence, operative skills, and transferable procedural-based learning were assessed.

**Results:**

Training with this cadaveric model resulted in a statistically significant improvement in self-reported confidence (*p* < 0.005) and prepared trainees for unsupervised bench work (*p* < 0.005). Respondents felt that the injected model allowed easier identification of vessels and ultimately increased the similarity to real-time operating. Our analysis showed it cost £10.78 and took 30 min.

**Conclusions:**

Perfusion of cadaveric limbs is both cost- and time-effective, with significant improvement in training potential. The model is easily reproducible and could be a valuable resource in surgical training for several disciplines.

Level of Evidence: Not ratable.

## Introduction

Traditionally, surgical training was centred on progressive operative practice under the supervision of a seasoned surgeon [1]. However, an increasing focus on patient safety, cost containment, and working hour restrictions has resulted in a perceived deficit in the training of junior surgeons [2–4]. Consequently, concerns have been voiced that some of these factors are negatively affecting operative caseloads, trainee confidence, and potentially undermining trainee comfort with independence in an operating environment [4–7].

An array of alternative pedagogic teaching methods has evolved in the wake of this expectation gap [8–14]. Virtual reality flap dissection has been mooted, but may be unrealistic and therefore have limited practical application [10] despite success in other specialities [15, 16]. Bench models are often versatile, reusable, and cost-effective [17]. They have also been objectively shown to improve basic surgical skills in novice trainees [18]. However, their role lies not in simulating a free flap procedure in its entirety but in the teaching of basic microsurgical skills in early training [10]. Thus, alternatives such as anaesthetised live animals have been previously popular as they facilitate microvascular flap-raising on animate, physiological tissue. Current UK legislation coupled with the need for specialised staff to oversee the animals has resulted in high cost of this modality. They also lack direct comparison with human tissues and anatomy which limits the transferable skill acquisition of the simulation [2, 11, 18, 19].

Human cadavers offer an alternative that benefits from accurate anatomy. However, it is not without its limitations which include high cost, limited availability, and use [10, 11, 20, 21]. Despite these issues, the utility of surgical rehearsal on cadavers is steadily increasing in reconstructive surgery [22–28] and other surgical disciplines alike [18, 20, 21, 29–34]. Conventional cadaveric dissection allows the trainee to familiarise themselves with gross anatomy of the flap and pedicle. However, it can prove difficult to both visualise and simulate the dissection of smaller vessels in a bloodless cadaver [22, 26].

Bloodless cadaveric dissection may be one factor that reduces the realism and therefore the transferable skills gained from cadaveric flap-raising. We therefore sought to enhance skill acquisition and improve the fidelity of such models by introducing a blood substitute to cadaveric dissection in a cost-effective, reproducible, and reliable protocol.

## Materials and methods

A review of current literature and local expertise were combined to form a study design protocol; optimal gelatin concentrations were established, as well as irrigation and injection methods on embalmed and fresh limbs. The enhanced fresh cadaver was then trialled and compared to conventional fresh cadaveric tissue during a preliminary study with local trainees. The protocol was then refined prior to its incorporation into our institutes’ cadaveric hand trauma course for further assessment. This study presents the evaluation of the finalised protocol. The total cost of supplies as well as time required in order to enhance a single cadaveric limb were recorded and summed.

All dissections were performed in the anatomy facility at our institute. Cadaveric limbs were donated according to the Anatomy Act 1984 (as amended by the Human Tissue (Scotland) Act 2006). Ten additional fresh/frozen upper limbs were acquired from ScienceCare (Phoenix, AZ, USA). Fresh limbs were maintained in a frozen state (− 7 °C) before use. Limbs were thawed at 14 °C, 12 h prior to dissection, and kept refrigerated between uses. Anonymity was protected throughout.

### Protocol

Upper limbs were amputated proximal to the cubital fossa. Fresh limbs were inverted and massaged to promote irrigation of fresh blood. All limbs were mechanically irrigated to remove blood, clot, or debris that may interfere with circulation. A 5FG-8FG 30-cm intermittent catheter was inserted into the brachial artery (BA), passed through the radial artery (RA) and then the ulnar artery proximal to the level of the wrist crease in order to physically dislodge any adherent thrombi. The catheter was then infused with 5-ml heparin (1000 units per 5 ml); as it was withdrawn from each vessel, warm tap water was infused until venous return or backflow was free from clots or debris. The volume of water required for each limb varied. All of the limbs were inverted and massaged to promote drainage of intravascular contents.

Following irrigation, the limbs were wrapped in a protective plastic sheath and submerged in a hot water bath (40 °C) to alleviate pressure points and promote the spread of the gelatin solution prior to setting. The gelatin solution was made to a concentration of 30 g/l with water and heated to 40 °C. Crimson red gouache paint was added until the desired colour was achieved to mimic blood. The limbs were manually injected with the gelatin solution via syringe and catheter as per the irrigation technique. Leaking vessels at the amputation site were clamped and injection continued until backflow from the BA was observed. The BA was subsequently tied with suture material and injection continued until adequate filling was achieved. Finally, the limbs were transferred to a refrigerated room (14 °C) with the syringe left raised above the limb to allow further infusion via gravitational feed whilst the gelatin solution set. The required volume of gelatin varied between 70 and 160 ml and was dependent on limb size and volume of leakage from open vessels prior to clamping.

### Qualitative evaluation

During 2016 and 2017, the injected limbs were evaluated during a hand trauma course, which is a 2-day training course (once per annum) for microsurgical flap reconstruction (day 1) and fracture fixation (day 2). A panel of plastic and orthopaedic surgeons attend and train on the course. Data was collected solely during the first day. Two or three trainees were assigned to one cadaveric model with one instructor to assist and supervise the reconstruction of fabricated hand injuries. This ratio allowed for adequate hands-on experience with the injected model for all participants. Procedures were carried out in a standard theatre manner with microsurgical instrumentation provided to enhance the realism. Prior to cadaveric dissection the course participants completed an anonymised questionnaire consisting of a self-evaluated scoring system (1 = not at all, 10 = completely) assessing “confidence,” “supervised comfort,” and “unsupervised comfort” with regard to flap-raising. At the end of day 1, respondents completed a post-dissection questionnaire consisting of the same parameters. The post-dissection questionnaire had an additional Likert scale (1 = strongly disagree, 10 = strongly agree) assessing respondents’ perception of the model’s characteristics, utility, and role within surgical training. During the course, photographs were taken to further illustrate the physical characteristics of the model.

The pre- and post-dissection confidence, supervised, and unsupervised comfort were analysed using a Wilcoxon matched paired test. A two tailed *p* value < 0.005 was considered significant. All other aspects of the questionnaire were analysed using descriptive statistics.

## Results

### Physical characteristics

**Fig. 1:**
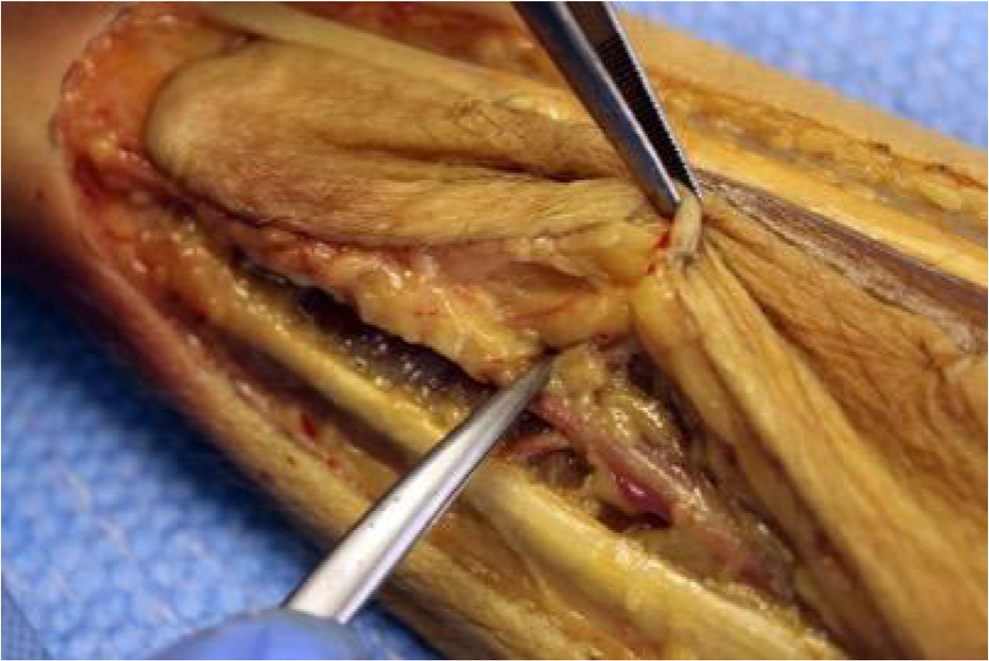
Medial perspective of radial forearm flap dissected in an injected fresh cadaveric limb. The radial artery and its accompanying venae comitantes are both turgid and stained. A small septocutaneous perforator rising up to supply the overlying skin

**Fig. 2:**
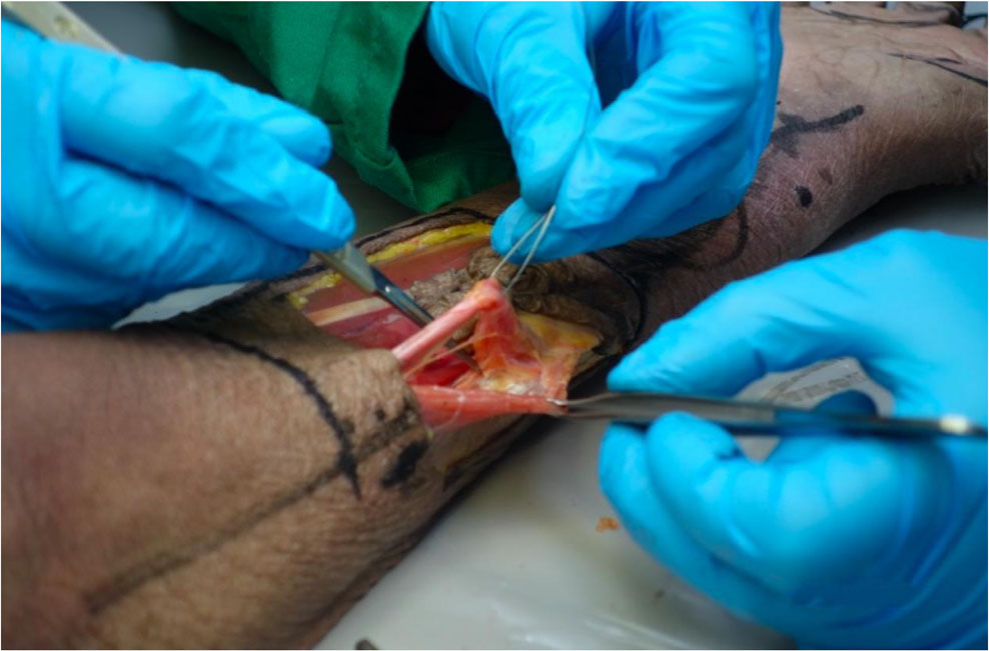
Radial forearm flap dissected in an injected fresh tissue cadaveric limb. The radial artery can be well visualised and dissected. A clear surgical field is maintained throughout

**Fig. 3:**
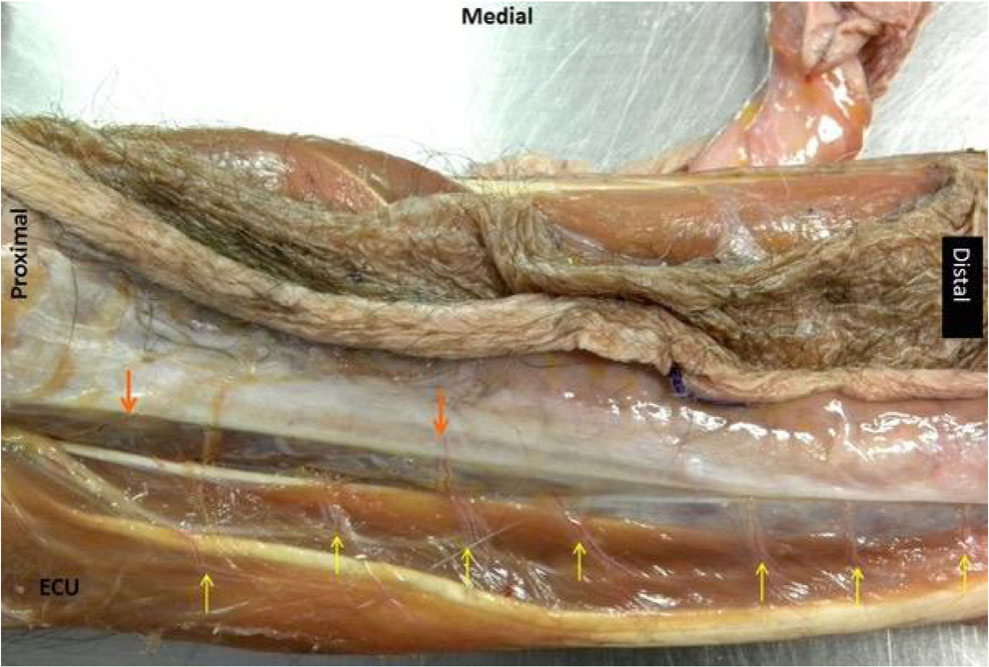
Lateral perspective of the dorsal aspect of a right injected fresh frozen forearm. The flap dissected is the posterior interosseous flap. Note that the posterior interosseous vessel is not visible; however, the septocutaneous (orange arrows) and musculocutaneous (yellow arrows) supplying the extensor carpi ulnaris (ECU) can be easily visualised and traced back to the major vessel. Indicators of medial, proximal, and distal aspects of the limb have been included for orientation

**Fig. 4:**
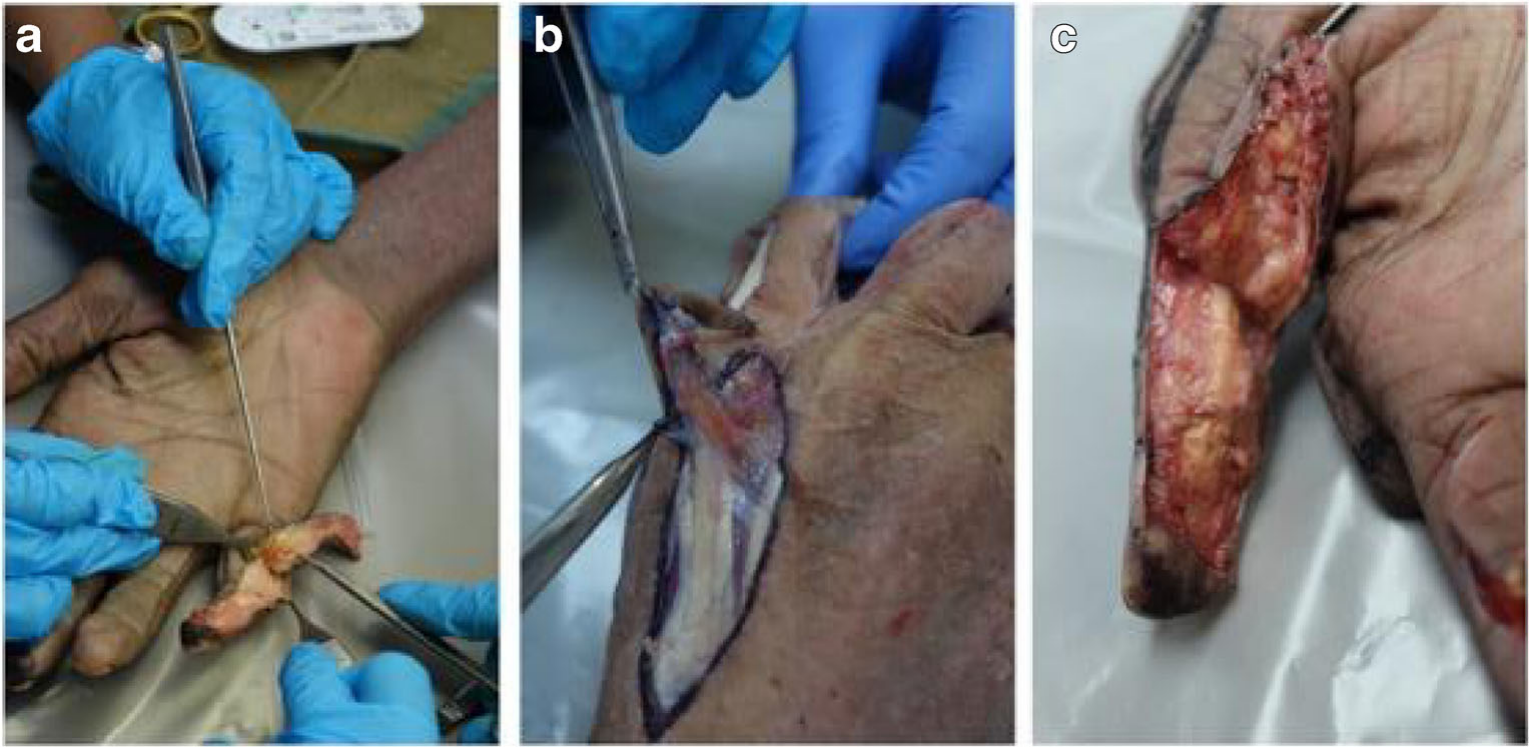
Flaps dissected in the hand included Venkataswami (**a**), Quaba (**b**), and Moberg (**c**)

Cadaveric tissue with the addition of gelatine injection provided realistic, pliable tissues which allowed for a range of flaps of varying difficulties to be carried out in the forearm (Figs. 1, 2, and 3) and hand (Fig. 4a–c). Vessels were turgid and stained appropriately, providing contrast from surrounding subcutaneous tissue and facilitated perivascular dissection. Small calibre vessels in the dorsal and volar forearm as well as distal hand were well and consistently filled in order for flaps of varying difficulties to be completed and the vasculature architecture studied (Figs. 1, 2, 3, and 4a–c). There were no instances of extravasation or aberrant tissue staining, thus allowing for a consistent clear surgical field.

### Cost and time analysis

The total cost to enhance a cadaveric limb was estimated at £10.78. The cost of reusable apparatus (catheter and syringe) was £7.87. The cost of non-reusable equipment was £2.91. This estimation takes into account the maximum recorded volume of gelatin solution required (160 ml of 3 g/100-ml gelatin concentration at a cost of 2.8 pence per gram), one 15-ml gouache paint tube supplying nine limbs (£5.00 per 15 ml tube), and 1000-unit heparin (£11.11 per 5 ml vial, 1000 units/ml). These figures do not account for facility fees, staff fees, or cost of procuring cadavers which vary according to institute. A total of 30 min of time is required to prepare a single limb.

### Qualitative evaluation

Fifty participants operated on 20 fresh-injected limbs. Participants were randomly allocated to each limb. Forty-four participants (88%) completed the survey; six participants did not wish to take part in the study. Respondents’ experience ranged from core surgical trainee 1 to specialist trainee 7 as well as international trainees whose exact training level cannot be defined in the British system; mentoring consultants were also asked to complete the questionnaires. Thirty-two respondents (72.7%) had previously been taught surgical techniques using cadavers. Furthermore, 26 respondents (59.1%) were already familiar with flap reconstruction in a clinical setting.

The median and interquartile range for trainees’ responses can be seen in Table 1. Trainees felt that the model was suitable for flap-raising simulation and was in agreement with all aspects of the questionnaire. Specifically, respondents felt that the injected model would improve future training (9.5 (8, 10)) and that it was superior to non-injected models (9 (8, 10)). Most importantly, the model was scored highly with regard to whether it should be integrated into both flap-raising courses (9.5 (8, 10)) and the surgical training curriculum (9 (8, 10)). Open-ended responses were positive with regard to the model. The most common positive feedback was that trainees felt that the model provided easier identification of vessels, realistic handling, and further added to the benefits of fresh tissue dissection. Constructive criticism suggested that the veins were not adequately filled in some specimens and pulsating vessels would have been appreciated.

**Table 1:** Course participants’ response regarding the utility and role of the enhanced cadaveric model

	Question	Median agreement level (interquartile range) (*n* = 44)
Perceived utility	This injected model facilitated the learning of flap anatomy and procedure	9.00 (8.00, 10.00)
	This injected model promotes surgical dexterity and level of skill needed for tissue dissection	9.00 (8.00, 10.00)
	This model allows for immediate feedback on surgical technique and decision-making	8.50 (7.25, 10.00)
	This model was a true simulation of the conditions of live surgery	8.00 (7.00, 8.00)
Role	This model would improve future training	9.50 (8.00, 10.00)
	Injected models are superior in training than cadaveric models without injection	9.00 (8.00, 10.00)
	This injected model should be integrated into all areas of the plastic surgery training curriculum	9.00 (8.00, 10.00)
	Training with this injected model should be integrated into flap raising training courses	9.50 (8.00, 10.00)
	This model improved my confidence and learning experience	9.00 (8.00, 10.00)

Cadaveric hand trauma course results: respondents’ level of agreement regarding the perceived utility and the role of the injected model. Data presented as median (interquartile range) on a Likert scale (1 = strongly disagree and 10 = strongly agree). *n* = total number of respondents

**Fig. 5:**
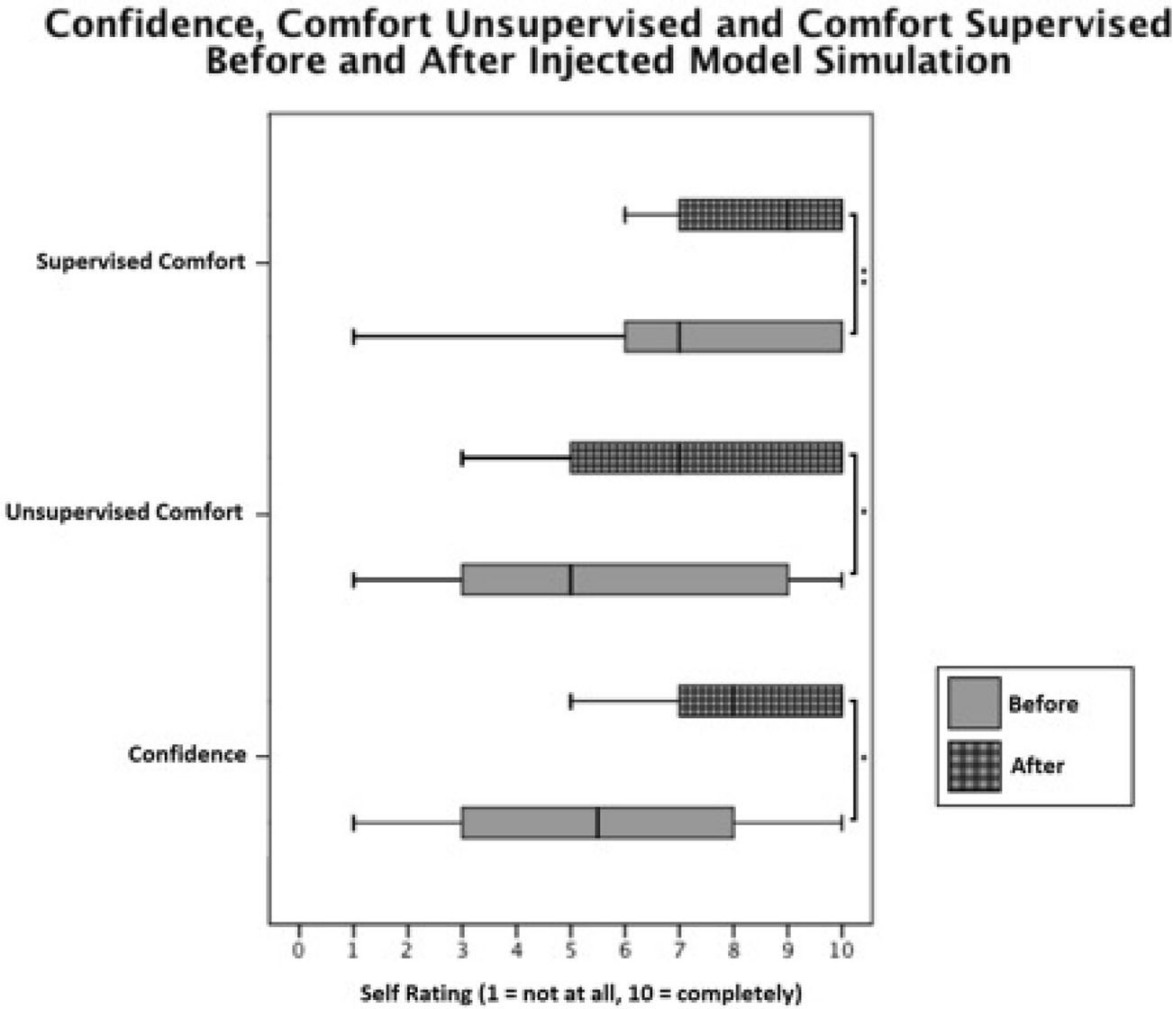
Box and whisker plots of self-rated (1 = not at all, 10 = completely) confidence, comfort unsupervised, and comfort supervised before and after cadaveric course simulation with the injected cadaveric model. * *p* < 0.005 (Wilcoxon paired test); ** *p* < 0.05 (Wilcoxon paired test). Box plots show median, 25 and 75% percentiles, as well as maximum and minimum ratings. *n* = 44 participants

A Wilcoxon matched paired test (Fig. 5) showed that trainees (*n* = 44) experienced a statistically significant rise in confidence following the use of the injected model (*Z* = − 3.76, *p* < 0.005). The median (IQR) pre-confidence and post-confidence were 5.5 (3.0, 8.0) and 8 (7.0, 10.0), respectively. However, five respondents did not note a change in their confidence. There was also a significant rise in unsupervised comfort (*Z* = − 3.19, *p* < 0.005). There was an observed rise in supervised comfort (*Z* = − 2.69, *p* < 0.05).

## Discussion

Under current conditions, there is widespread concern that trainees are less experienced in theatre. In this study, trainees were provided with an opportunity to use an enhanced cadaveric model in order to tackle a variety of procedures of varying complexity encountered in a clinical setting. This furthers the trainees’ experience in a safe environment whilst not affecting true operating time or patient outcomes. Not only have we demonstrated a cheap and feasible method of enhancing cadaveric training, we have demonstrated one which is well received and beneficial to trainees. Enhanced cadaveric training should be considered a useful adjunct to the plastic surgery curriculum as well as training courses.

A technique for cadaveric gelatin infusion for the purpose of surgical education has been previously described involving the infusion of gelatin solution via gravitational feed [35]. They have only commented on the filling of gross vessels. Our group felt that manual injection provides pressures which promote the filling of small-calibre, high-resistance vessels. The gelatin concentration may also play a role in ensuring vascular filling [36]. If the concentration is too high, the gelatin may set prior to even distribution within the vasculature. Previously, higher gelatine concentrations have been suggested [35–39]. We believe that this study’s concentration (30 g/L) optimises the perfusion of smaller peripheral vessels. Objective means of assessing adequate gelatine filling were not employed; however, a range of flaps could be dissected, with filling down to the arteriole networks only visible under under loupe magnification (×4); this was taken as sufficient evidence that the technique was effective. One study has shown that gelatine solution (100 g/L) injected into fresh cadaveric limbs results in the filling of vessels less than 0.01 mm [39]. In future studies using the described injection protocol, measurement of the smallest vessel calibres should be employed.

Several materials have been used to fill and facilitate the dissection of blood vessels. One study compared characteristics for commonly used injectable materials for fresh upper limb tissue dissection [39]. These included latex, silicone, araldite F, Batson’s no. 17, and gelatine. In our experience, several injectable products such as Araldite F and Batson’s no. 17 are strong and offer good penetration of a given vasculature; however, they are not suitable for high-fidelity surgical simulation owing to their properties such as lack of flexibility and increased vessel fragility. Coloured latex has been used but the extent of vessel penetration cannot be well controlled resulting in colouration of tissues beyond the vasculature, impeding on fidelity [37]. The high viscosity of latex can result in suboptimal filling; it also requires lengthy preparation time and solidification can require immersion in formalin solution which may affect tissue and blood vessel integrity [37, 39]. However, latex only costs £1.66/100 ml [39]. Silicone provides excellent penetration; however, it is expensive (£117.09/100 ml) and can increase vessel fragility [39]. Whilst all injectable fluids are suitable for vessel injection, the choice of which injectable and its associated characteristics one should use is goal specific. For surgical flap dissection courses, gelatine may offer superiority over alternatives, which our department favours because of ease of preparation, vessel fidelity, and penetration at an acceptable cost.

Qualitative evaluation suggested that respondents were in agreement that the injected model was superior to non-injected fresh cadaver models (Table 1). Although not all trainees had prior conventional cadaveric experience, fresh cadavers promote gross anatomical learning [20, 21, 34] and help trainees develop an insight into technical procedural steps [20, 33]. The injection of vessels with gelatine provides a more precise interpretation of the fine vascular of a given flap [36–38]. The contrast between vessels and perivascular tissue in the injected model allows for easy differentiation and tracing of perforators to promote an exact knowledge of flap anatomy essential for flap design and elevation. Furthermore, the use of gelatine optimises the dexterity required for dissection by providing vessels with the structural integrity and visual cues required to facilitate perivascular dissection [38].

Despite course respondents rating all aspects of the questionnaire highly, the lowest rated aspect was whether “the model was a true simulation of the conditions of live surgery.” There has been recent focus on the reconstitution of the post-mortem circulation in order to provide a closer approximation to living tissue [19, 22, 23, 28, 40, 41]. Garrett [19] pioneered the development of an arterial circulation intended for endovascular procedures. Although innovative, its limitations include thrombi/debris blocking the tubing system, the lack of physiological flow from arterial to venous circulation, and massive oedema formation that can cause considerable tissue distortion, rendering a model unusable for up to 24 h. Subsequent models have not adequately addressed these limitations [22, 23, 28, 40, 41]. Additionally, the use of blood substitutes can cause profuse bleeding and tissue staining which may limit the educational benefit and extend the dissection time [28, 40]. The cost of sophisticated models which bypass some of the limitations of previous models has been estimated at $1262.55 [41], not accounting for facility fees or cost of procuring cadaveric material.

In comparison, our model has minimal cost, does not require specific expertise to maintain it during the simulation, does not affect tissue characteristics, and ensures a clear surgical field for the trainee learning the fundamentals of flap surgery. Where post-mortem circulations cannot realistically mimic bleeding and physiological circulation, an injected model could be more appropriate for enhancing simulation.

A statistically significant increase in mean confidence, unsupervised, and supervised comfort was noted. Whether this is due to gelatine injection, cadaveric training or coaching remains unclear; in reality, it is a combination of a more lifelike specimen for enhanced dissection under the tutelage of experienced faculty. We believe our model can only aid in building trainee confidence and comfort with procedures, as we provide additional visual cues and realism to enhance the dissection experience. The use of a control group would clarify this; however, it was deemed inappropriate to offer only half of paying course participants an enhanced cadaver. However, it should be noted that an initial local pilot study comparing injected and non-injected cadavers was undertaken of this nature in order to establish validity of an injected model. Trainee feedback suggested that an injected model was favoured as a more useful adjunct to plastic surgery training when compared with its conventional counterpart with regard to the identification and characteristics of major arteries and perforating arteries, facilitating the learning of flap anatomy, as well as improving trainee confidence. Cadaveric simulation has previously been shown to improve operative confidence in inexperienced surgeons [20, 25, 29, 30]. This is the first report of an increase in unsupervised comfort following cadaveric training. It is logical to consider the importance of self-confidence and comfort in the theatre. Confident trainees may have an increased willingness to participate in more challenging complex cases; increasing operative confidence may promote composure and decision-making in challenging situations, minimise fatigue, and it has been suggested to be a major influence in acquisition of expert performance [42–44]. However, it should be noted that falsely inflated confidence could have detrimental effects on patient outcomes.

### Limitations

This study has several limitations. Firstly, a validated instrument was not used in this study. This was considered a time- and labour-intensive process beyond the scope of this initial study. As an ongoing project, it is the aim to improve and validate the questionnaire used. In future courses, we hope that all participants will perform dissection on injected cadavers as well as conventional cadavers in order to provide a true comparison of models. Furthermore, consultant experts were included in cadaveric course analysis, for whom a rise in confidence or comfort may not occur based on the scale used in the questionnaire; however, we desired expert feedback as well as trainee feedback; thus, it was felt pertinent to include consultants in the study. It should be noted, previously, that it has been shown that there is a positive correlation between plastic surgery trainee seniority and mean confidence increase after cadaveric flap simulation [25]. The proposed reason for this is that seniors have completed the necessary prerequisite training to benefit in a different way from cadaveric simulation. It would be interesting to compare consultant and trainee feedback with regard to confidence, comfort, and model characteristics. Due to the number of respondents included in this study, this comparison was not warranted.

### Conclusion

This study demonstrates that injected cadaveric models were perceived as a useful adjunct that could improve future microsurgical training. When we consider the additional low cost, reliability, and ease of preparation of this model, it would seem reasonable to replace conventional cadaveric models in order to improve the simulation of raising free flaps. We suspect that this model may have applications in other surgical disciplines. We aim to incorporate this model into future courses for further assessment and produce alternative cadaveric models suitable for flaps beyond the upper limb.
